# Buprenorphine vs. morphine: impact on neonatal opioid withdrawal syndrome (NOWS) outcomes in a single center retrospective study

**DOI:** 10.1038/s41372-024-02046-7

**Published:** 2024-07-13

**Authors:** Saminathan Anbalagan, Victoria Anderson, Michael T. Favara, Daniela Stark, David Carola, Kolawole Solarin, Susan Adeniyi-Jones, Walter K. Kraft, Zubair H. Aghai

**Affiliations:** 1 Deparment of Pediatrics/Neonatology, Thomas Jefferson University/Nemours, Philadelphia, PA USA; 2 Deparment of Pediatrics/Neonatology, University of South Alabama, Mobile, AL USA; 3 Deparment of Pediatrics/Neonatology, ChristianaCare Hospital, Newark, DE USA; 4 Department of Pharmacology, Physiology, and Cancer Biology, Thomas Jefferson University, Philadelphia, PA USA

**Keywords:** Therapeutics, Addiction, Outcomes research

## Abstract

**Objectives:**

To compare clinical outcomes for infants with neonatal opioid withdrawal syndrome (NOWS) treated with buprenorphine or morphine.

**Study design:**

Retrospective study of infants born ≥35 weeks’ gestation and admitted to the NICU for NOWS treatment between 2011 and 2022. Length of treatment, length of stay in the hospital, and the need for secondary medications were compared between buprenorphine and morphine treated neonates. Multiple regression analysis was performed, adjusting for baseline differences and confounders.

**Results:**

417 neonates were treated with morphine and 232 with buprenorphine. The buprenorphine group had shorter treatment days [−10.8 days; 95% CI: −8.08 to −13.53] and shorter hospital stay [−11.8 days; 95% CI: −8.83 to −14.78]. The buprenorphine group was no more likely to receive phenobarbital or clonidine (26% vs. 29%).

**Conclusion:**

In this large single-center study, buprenorphine was associated with shorter lengths of treatment and hospital stay in the treatment of NOWS compared to morphine.

## Background

Neonatal opioid withdrawal syndrome (NOWS) is seen in infants with in-utero exposure to opioids who demonstrate at least 2 of 5 signs: excessive crying, fragmented sleep, tremors, increased muscle tone, and/or gastrointestinal dysfunction [1]. The incidence of NOWS has risen over the past few decades, mirroring the opioid epidemic’s escalation [2, 3]. NOWS is associated with significant health care costs, exceeding $2.5 billion annually [4]. These findings underscore the urgent need for effective strategies to prevent and manage NOWS, not only to mitigate the long-term health consequences for affected infants but also to alleviate the financial burden on healthcare systems and society.

While non-pharmacological treatment is recommended by AAP as the first-line therapy [5], pharmacological treatment with opioids remains a cornerstone of NOWS management when non-pharmacological measures fail. Oral morphine and methadone remain the most prescribed first-line medications for treating NOWS in the United States [6]. However, there has been growing interest in using sublingual buprenorphine as an alternative treatment approach [7, 8]. Buprenorphine has demonstrated improved efficacy compared to other opioids used to treat NOWS in randomized controlled trials [9, 10], retrospective chart reviews [11], and in the context of quality improvement projects [12]. The efficacy advantage of buprenorphine has been supported by meta-analysis as well [13]. However, the effects from smaller phase I-III randomized controlled trials (RCTs) [9, 10, 14] and retrospective reviews with varying degrees of adjustment for confounding variables [11, 15] are limited by their sample sizes.

Our neonatal intensive care unit (NICU) changed from morphine to buprenorphine as first line therapy in November 2017. This switch in institutional protocol provided a natural experiment to conduct this cohort analysis. The main objective was to evaluate the impact of these treatments on the length of treatment and length of hospitalization, with the secondary outcome being the proportion of infants necessitating adjunctive pharmacotherapy.

## Methods

### Study design and population

This is a retrospective study of infants born over 11 years, spanning from January 1^st^, 2011, to July 30^th^, 2022. The study population consisted of infants born at a gestational age (GA) of ≥35 weeks and admitted to a level 3 NICU in Philadelphia for pharmacotherapy and medical management of NOWS. Institutional protocol stipulated all infants with NOWS that needed pharmacotherapy be admitted to the NICU. Infants who were born preterm (<35 weeks’ gestation) or enrolled in any clinical trial were excluded from the study. This study was conducted in accordance with the ethical principles outlined in the Declaration of Helsinki. The study was approved by the Institutional Review Board (IRB) of Thomas Jefferson University, PA, and the IRB waived the informed consent due to the retrospective nature and minimal risk posed by this research. Patient confidentiality was strictly maintained throughout the data collection and analysis process. De-identified patient information was stored in a centralized database in the institution. Infants admitted to the NICU with a diagnosis of NOWS were tracked as part of optimization of clinical care and this was primarily used to identify the subjects for this study. All had a urine and/or meconium drug screen done.

### Severity and treatment of NOWS

**Fig. 1:**
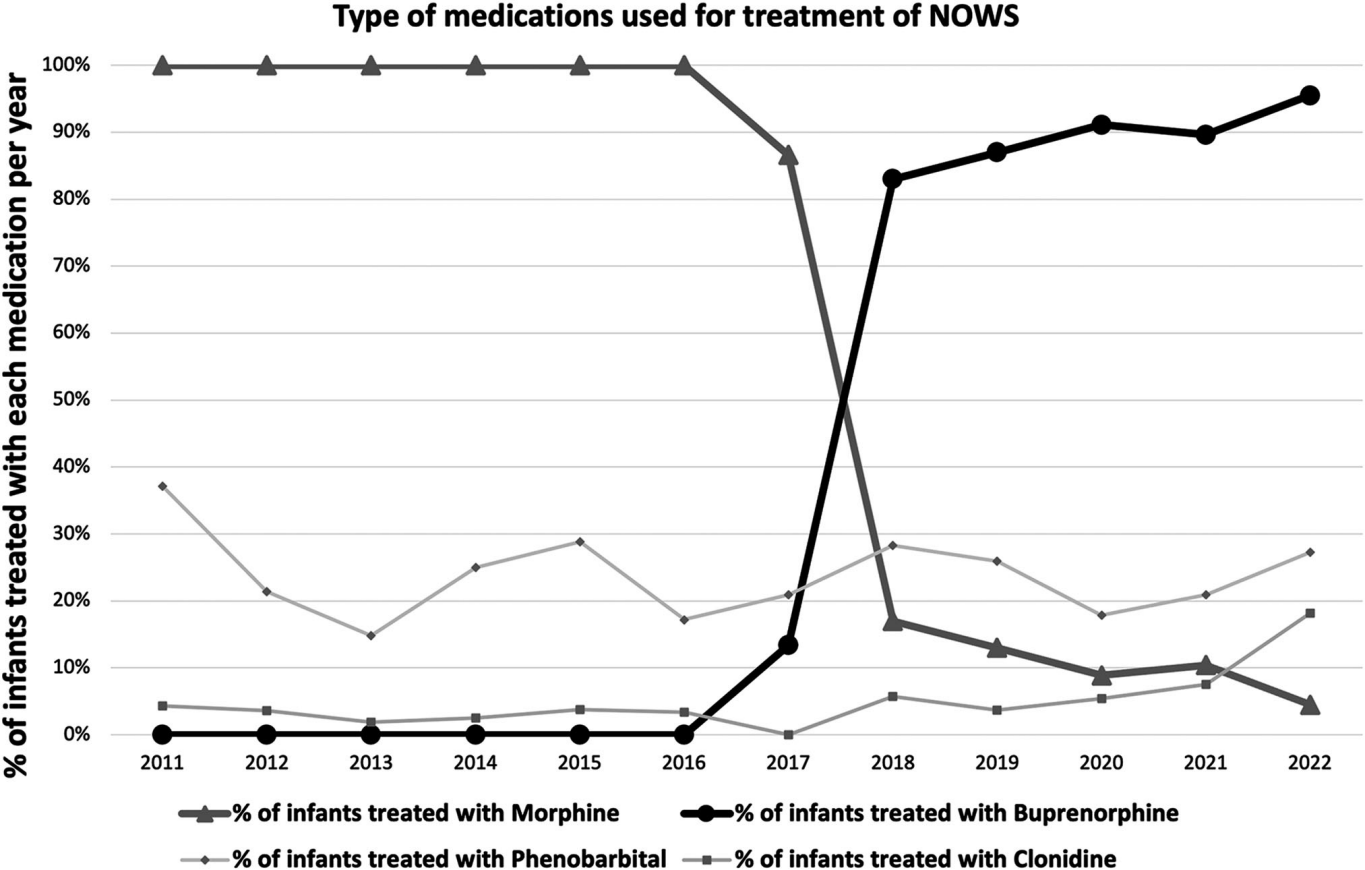
Trends in the use of pharmacotherapy for NOWS during this study period. Triangle represents percentage of infants treated with morphine; dot represents buprenorphine; diamond represents phenobarbital, and square represents clonidine.

The MOTHER NAS tool, a modified Finnegan scoring system, (19 criteria, score range 0 to 40) [16] was used to assess the severity of withdrawal. The institutional policy included a multidisciplinary approach for all infants with NOWS, primarily maximizing the non-pharmacological care, cohorting infants with NOWS, encouraging parental involvement as much as possible, and facilitating breastfeeding when the mother was abstinent or enrolled in a methadone maintenance program. Only those that had symptoms uncontrolled with these measures and meeting the institutional protocol were treated with medications. Pharmacotherapy was initiated with a single score ≥12 or three consecutive scores equal to a sum of >24. Before November 2017, all infants with NOWS requiring pharmacotherapy were treated with morphine as the primary medication (Fig. 1). After November 2017, the institutional protocol shifted towards sublingual buprenorphine as the first line agent, except for neonates <36 weeks, continuation of therapy begun at outside hospital, maternal request, NPO status, or other contraindications for sublingual buprenorphine (e.g. orofacial anatomical defects like cleft lip/palate, orogastric diseases like diffuse oral thrush or mucositis, severe illness requiring intubation) - in such situations, morphine was used. If the neonate had received both buprenorphine and morphine at any point during the hospital course, they were excluded from the study. A standardized protocol was followed for the escalation and weaning of both medications (see supplement). When symptoms were not well controlled with the opioids, phenobarbital was added as the second-line therapy and clonidine as the third-line therapy. Even though a departmental protocol existed to manage these adjunct medications, treating physicians could modify them according to the patient’s needs.

### Data collection

The following neonatal variables from the medical records were collected: gestational age at birth (in weeks of completed gestation), sex (as determined at birth), race/ethnicity (as noted in the medical record and reported by parents – black, white, ‘others,’ and Hispanic), method of delivery, and 5-minute Apgar score. Birth anthropometrics (birth weight, head circumference, and length, reported in S.I. units) and small for gestational age (SGA) status were determined using the Fenton growth curve [17].

The following maternal data was also obtained: age (in years), enrollment in a methadone maintenance program (yes/no), methadone dose (in milligrams per day), type of opioid use (heroin, fentanyl, suboxone, oxycodone; reported as yes/no), use of prescription medications such as benzodiazepines and selective serotonin reuptake inhibitors (yes/no), tobacco use (yes/no), and other illicit substances intake (cocaine, tetrahydrocannabinols or THC, and phencyclidine or PCP). The number of drugs of abuse (non-prescribed opioids and illicit substances as mentioned above) was noted, while the polysubstance use was recorded as yes/no if the mother had used any opioid plus any illicit substances or prescribed medications as noted above.

### Outcomes

The primary outcome of this study was to investigate the Length of Treatment (LOT) and Length of Stay (LOS) in the hospital among infants with NOWS. LOT was calculated from the first day of treatment until the last day of any pharmacotherapy for NOWS, including as-needed opioids. None of the infants were discharged home with medications. LOS was calculated from the day of birth until the day of discharge or the day of the transfer to another institution. The secondary outcome of interest was the need for adjunct medications for NOWS, i.e., phenobarbital or clonidine.

### Statistical analysis

Descriptive statistics were used to summarize the demographic and clinical characteristics of the study population. Continuous variables were reported as medians with interquartile ranges based on their distribution. Categorical variables were presented as frequencies and percentages. Comparative analyses between the two groups were performed using appropriate statistical tests, such as chi-square tests or the Mann-Whitney U test. Linear and logistic regression analyses were performed to adjust for known confounders such as SGA status, and benzodiazepine exposure, baseline differences, and year of birth (in quarters). Findings from adjusted analyses are reported as relative risk differences and odds ratios along with 95% confidence intervals. All statistical tests were two-sided, and significance was set at a standard *p* < 0.05. IBM SPSS statistical software version 23.0 (IBM Corp., Armonk, NY, USA) was used to perform these statistical tests.

## Results

A total of 649 infants were eligible. Total patient characteristics are as follows: 332 (51%) were assigned male sex at the time of birth, 377 (58%) were white race, and 425 (65.5%) were born by vaginal delivery. The overall distribution of GA was 38 weeks (IQR: 37 to 39 weeks) with a median BW of 2.88 kg (IQR: 2.53 to 3.22 Kg), and of those, 153 (23.6%) were categorized as SGA. Nine infants (out of 649) were transferred to another institution for higher level of care not related to NOWS. Median maternal age was 30 years (IQR 27–33), and the majority were enrolled in a methadone maintenance program (509 or 78%) with a median daily dose of 125 mg (IQR 80–175 mg/day), and a significant portion of them had polysubstance use (438 or 67.5%).

Two hundred and thirty-two neonates (36%) were treated with buprenorphine. Table 1 describes the maternal and infant characteristics. There was a statistical difference in the gestational age between the two groups (*p* = 0.024) even though the median GA and IQR were the same across groups. Race/ethnicity was also statistically different (*p* < 0.001) between the two groups (race was an optional entry in our institution before 2017). The neonates that were treated with buprenorphine were also more likely to be SGA (*p* = 0.01). The mothers of neonates that were treated with buprenorphine were older in age, smoked tobacco, used heroin and THC frequently, had a higher rate of polysubstance abuse of up to three drugs, and were less frequently enrolled in a methadone maintenance program. All of these findings were statistically significant with *p* < 0.001.

**Table 1:** Neonatal and maternal demographics and clinical characteristics (median, IQR).

	Morphine group (*N* = 417)	Buprenorphine group (*N* = 232)	*p* value
*Neonatal characteristics:*			
Gestational age in weeks	38 [37,39]	38 [37,39]	**0.024**
Male sex, *n* (%)	219 (52.5%)	113 (48.7%)	0.35
Race/ethnicity, *n* (%):			
White	216 (51.8%)	161 (69.4%)	**<0.001**
Black	60 (14.4%)	49 (21.1%)	
Hispanics	19 (4.6%)	11 (4.7%)	
Unknown	120 (28.8%)	9 (3.9%)	
Method of delivery, *n* (%):			
Vaginal	275 (65.9%)	150 (64.7%)	0.21
C-Section	137 (32.9%)	82 (35.3%)	
5-minute Apgar score	9 [9,9]	9 [9,9]	0.85
Birth weight in Kg	2.892 [2.55, 3.232]	2.829 [2.49, 3.149]	0.13
Birth head circumference in cm	33 [31.5, 34]	33 [31.5, 33.5]	0.71
Birth length in cm	48 [46.5, 50]	48 [46,50]	0.12
Small for gestational age, *n* (%)	85 (20.4%)	68 (29.3%)	**0.01**
*Maternal characteristics:*			
Age in years	29 [26,32]	31 [28,35]	**<0.001**
Enrolled in methadone maintenance program, *n* (%)	352 (84.4%)	157 (67.7%)	**<0.001**
Methadone dose in mg	120 [80, 170]	125 [70, 195]	0.41
Multiple drugs of abuse, *n* (%)	265 (63.5%)	173 (74.6%)	**0.004**
Number of multiple drugs	1 [0, 2]	1 [0, 3]	**<0.001**
Heroin use, *n* (%)	144 (34.5%)	98 (42.8%)	**0.038**
Cocaine use, *n* (%)	126 (30.2%)	73 (31.6%)	0.71
THC use, *n* (%)	66 (15.8%)	59 (25.5%)	**0.003**
PCP use, *n* (%)	15 (3.6%)	5 (2.2%)	0.31
Benzodiazepine use, *n* (%)	154 (36.9%)	66 (28.6%)	**0.031**
Smoking, *n* (%)	237 (56.8%)	178 (76.7%)	**<0.001**
SSRI use, *n* (%)	56 (13.4%)	27 (11.6%)	0.51

Bold indicates statistically significant finding.

Analysis with chi-square for categorical variables, Mann-Whitney U test for continuous variables.

*NOWS* Neonatal Opioid Withdrawal Syndrome, *SSRI* Selective Serotonin Receptor Inhibitors, *PCP* Phencyclidine, THC Tetrahydrocannabinols, *IQR* Interquartile Range.

**Fig. 2:**
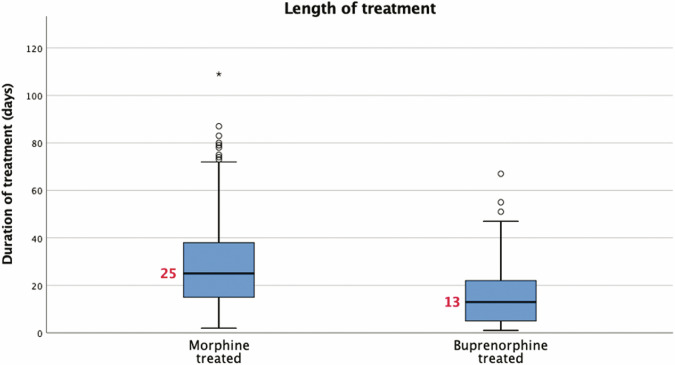
Box plot of length of treatment for infants with NOWS receiving morphine or buprenorphine in unadjusted analysis. Boxes represent interquartile ranges, horizontal lines inside the boxes & the red numbers represent medians, whiskers represent 5th and 95th percentiles, and asterisk/dots represent outliers.

**Fig. 3:**
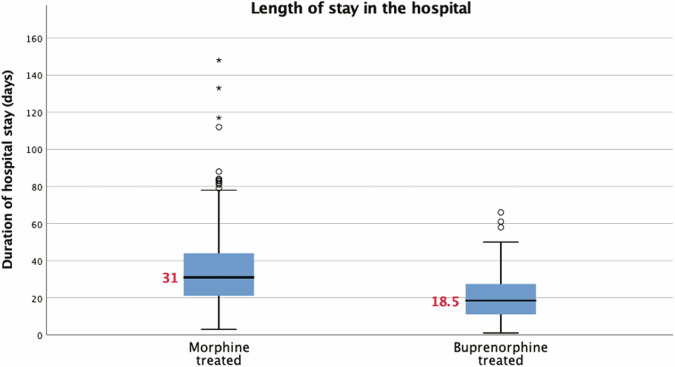
Box plot of length of stay in the hospital for infants with NOWS receiving morphine or buprenorphine in unadjusted analysis. Boxes represent interquartile ranges, horizontal lines inside the boxes & the red numbers represent medians, whiskers represent 5th and 95th percentiles, and asterisk/dots represent outliers.

The median LOT and LOS of the two groups are shown in Figs. 2 and 3. The LOT was significantly shorter in those neonates treated with buprenorphine compared to the morphine-treated group, a difference of 12 days in medians. Similarly, the LOS in the hospital was also significantly shorter in the buprenorphine-treated group, a difference of 12.5 days in the medians. These differences were found to be statistically significant in unadjusted analysis (*p* < 0.001), and this significance was maintained with a relatively tight confidence interval even after adjustment for confounders and baseline differences in the multiple regression analyses (Table 2). But the need for adjunct medications, phenobarbital and clonidine, were similar in both groups (Table 3). Of note, 87 (20.9%) of neonates were treated with morphine after November 2017.

**Table 2:** Neonatal outcomes – primary (median, IQR).

	Morphine group (*N* = 417)	Buprenorphine group (*N* = 232)	Risk difference (95% CI)	*p* value
Length of treatment in days	25 [15,38]	13 [5,22]	−10.8 (−8.08 to −13.53)	**<0.001**
Length of stay in the hospital in days	31 [21,44]	18.5 [11, 27.75]	−11.8 (−8.83 to −14.78)	**<0.001**

Bold indicates statistically significant finding.

Analysis performed by linear regression adjusting for race, GA, SGA status, infant’s year of birth, mothers age, maternal methadone, benzodiazepine, heroin, THC, tobacco, and polysubstance use.

Length of treatment reflects medications during the hospital stay.

*NOWS* Neonatal Opioid Withdrawal Syndrome, *IQR* Interquartile Range.

**Table 3:** Neonatal outcomes – secondary (*n*, %).

	Morphine group (*N* = 417)	Buprenorphine group (*N* = 232)	*p* value
Need for secondary medication for NOWS:			
Phenobarbital	105 (25.2%)	49 (21.1%)	0.24
Clonidine	15 (3.6%)	13 (5.6%)	0.23

Unadjusted analysis by chi-square test.

*NOWS* Neonatal Opioid Withdrawal Syndrome, *IQR* Interquartile Range.

## Discussion

This comparative analysis in a large cohort of mother-infant dyads revealed that neonates treated with buprenorphine had shorter treatment duration and hospital stay compared to those treated with morphine. No differences in the need for adjunct medications were noted between the two treatment groups. These findings corroborate the growing consensus regarding the effectiveness of buprenorphine in the management for NOWS.

Several researchers have noted a similar reduction in LOT and LOS like the results in this study [9, 10, 14, 15, 18]. Initial evidence from our institution in a phase I study suggested that sublingual buprenorphine was effective in controlling NOWS symptoms and was safe, well tolerated, and resulted in shorter treatment and hospitalization [14]. A subsequent double blinded RCT in 2017 comparing buprenorphine and morphine further established that buprenorphine treatment resulted in a significantly shorter LOT (13 days difference) and shorter LOS (12 days difference) without an increase in adjunct medications [10]. None of the infants in the current report were included in the clinical trials of buprenorphine in our institution. Moreover, comparative studies have identified buprenorphine as superior to methadone, showing comparable reductions in LOT and ~7 fewer hospital days [11]. Beyond RCTs and retrospective reviews, a QI initiative assessed the duration of therapy and length of hospital stay following implementation of buprenorphine for NOWS and noticed a 6-day reduction in LOT and 5.5 day reduction in LOS [12]. Eat, Sleep, and Console (ESC), a novel functional approach, along with buprenorphine implementation for NOWS, has also produced similar results [19]. These provide external validity to the findings of this study and are potential opportunities for reduced healthcare-associated costs as well.

Notably in this study, a shorter LOT and shorter LOS in neonates treated with buprenorphine was noted despite the low rate of maternal methadone enrollment and high rate of polysubstance abuse. This finding attests to the efficacy of buprenorphine in the management of NOWS from different types of opioid exposure. Supporting this, Taleghani et al. in 2019 reported a reduction of LOT and LOS by ~50% with the use of buprenorphine for chronic methadone exposure in-utero [15]. Similarly, another retrospective review of 360 infants exposed to a wide spectrum of opioids in-utero also revealed buprenorphine’s superiority to traditional opioids, reducing LOT by an average of 3 days and LOS by 2.8 days [18] It is postulated that these observations of shorter therapy duration and hospital stay with sublingual buprenorphine are possibly due to its pharmacodynamic and pharmacokinetic properties [20, 21]. It has a dual effect on the opioid receptors with a high binding affinity and a slower dissociation, in addition to high lipophilicity and sublingual route of administration. Additionally, buprenorphine’s extended half-life and reduced peak-to-trough ratio are also hypothesized to contribute to its efficacy [22].

This study has several strengths: this is the first known study to analyze such a large cohort of infants with NOWS, specifically with buprenorphine therapy. This study accounted for several well-known confounders, such as maternal benzodiazepines, SSRIs, polypharmacy, smoking, and infants’ SGA status. It is also comprised of a diverse population of infants who experienced NOWS from exposure to opioids both through medication-assisted treatment (MAT) and illicit drug use. Statistically and clinically significant differences in the outcomes of interest were also found, thus providing practical and applicable information.

However, certain limitations to this study must be acknowledged. The average length of stay in this study was longer than the reported national average of 22.2 - 23.8 days [23]. This discrepancy may be attributed to drifts in clinical practices, variations in the tapering protocols, and a patient population that was severely affected by the opioid epidemic [24]. The increase in the use of clonidine between 2020 and 2022, albeit a non-significant finding, can also be speculated to reflect the treating physician’s preference secondary to increased polysubstance exposure and severity of NOWS over time (Table 1). Additionally, social determinants may have resulted in a prolonged hospital stay. The data was collected over a period of 11 years, where practice changes such as implementation of algorithmic treatment may have affected the outcomes; however, the year of birth was accounted for in the regression analysis and the primary findings of this study remained significant. Moreover, ~21% infants were treated with morphine even after the switch in standard of care, implying the temporal effects may not be a critical factor for the outcomes noted. Also, this is the largest cohort published to date with a diverse population, and the findings are consistent with the results of the published RCTs. This study was conducted on maternal-infant dyads from a single center in a retrospective non-randomized method, which affects the generalizability of our results. The retrospective nature of this study introduces the possibility of inaccurate information and unidentified residual confounding variables. While morphine was used in situations where sublingual buprenorphine was contraindicated, the effects of severity of the underlying non-NOWS conditions cannot be excluded. Further, the omission of the type of feeding must be noted as this could significantly influence the primary outcomes; reports show that neonates fed with maternal breastmilk had less severe NOWS symptoms, decreased need for pharmacotherapy, and shorter length of stay [25, 26]. Lastly, despite the robust finding of improved short-term outcomes, the potential benefits of buprenorphine treatment for NOWS on long-term outcomes were not explored in this study.

In summary, these findings reinforce the position of buprenorphine in the literature as a preferred therapeutic agent for NOWS, with evident advantages in treatment and hospitalization durations compared to morphine. To extend the utility of these findings, future research should focus on prospective studies of infants with NOWS encompassing larger cohorts and longitudinal assessments to elucidate the full spectrum of buprenorphine’s impact, particularly on long-term neurodevelopmental outcomes. At the same time, a multidisciplinary and comprehensive approach to NOWS that involves family participation is key in improving the outcomes.

## Conclusions

In this retrospective analysis of a large single-center cohort of infants NOWS, treatment with sublingual buprenorphine was associated with significantly shorter treatment days and hospital stay compared to treatment with traditional oral morphine. The use of adjunct medications, specifically phenobarbital and clonidine, did not differ significantly between the buprenorphine and morphine-treated groups, suggesting similar efficacy in managing withdrawal symptoms.

## Supplementary information

Figure S1. Institutional protocol for management of pharmacotherapy for NOWS using buprenorphine.

Figure S2. Institutional protocol for management of pharmacotherapy for NOWS using morphine.

## Data Availability

Data will be made available upon reasonable request.
