# Supplementary figures and images for: Buprenorphine vs. morphine: impact on neonatal opioid withdrawal syndrome (NOWS) outcomes in a single center retrospective study
Source: J Perinatol. 2024 Jul 13;45(4):473–9. doi: 10.1038/s41372-024-02046-7 (PMC12069082; doi:10.1038/s41372-024-02046-7)

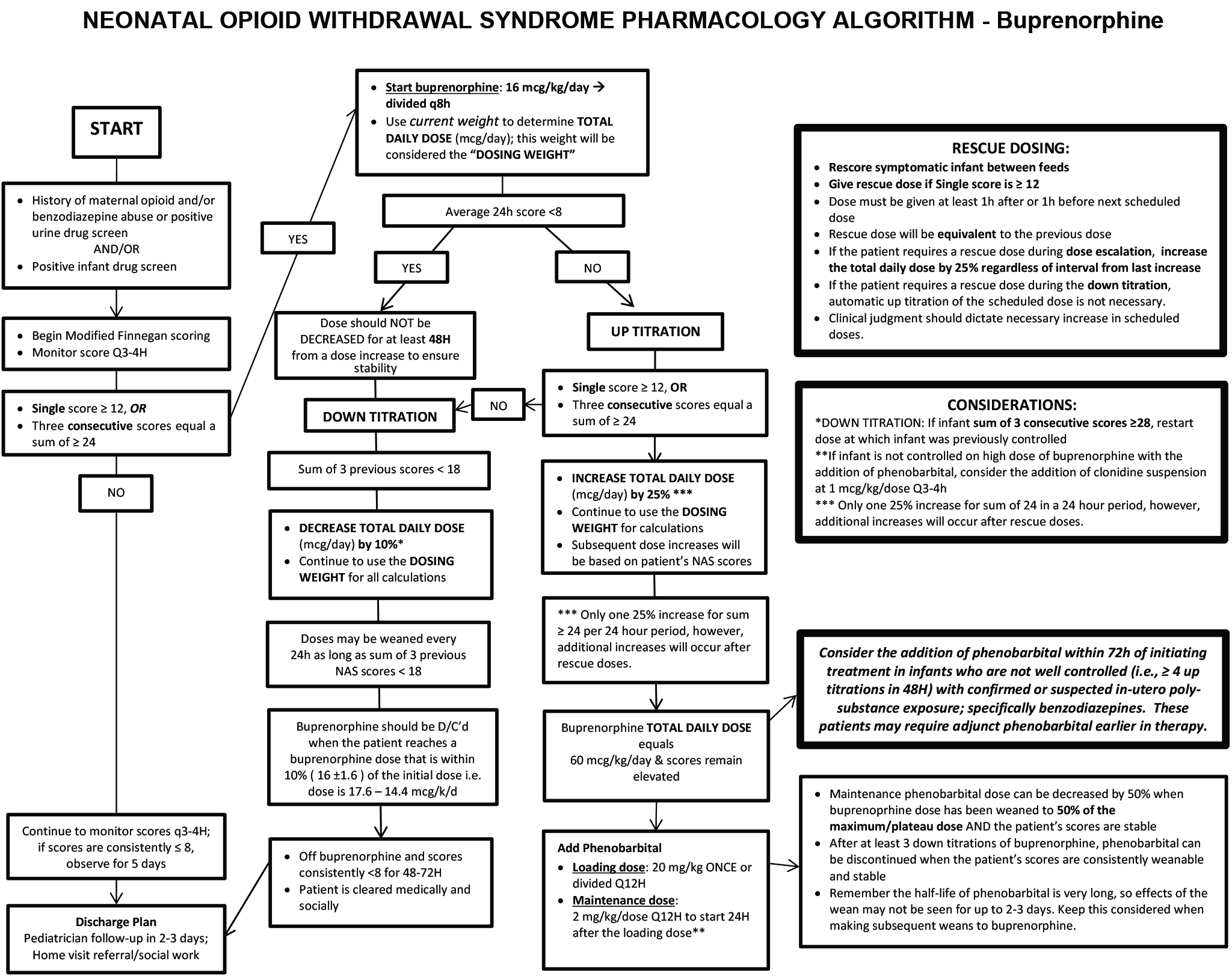

Supplement: Supplementary file 1 — Figure S1. Institutional protocol for management of pharmacotherapy for NOWS using buprenorphine. [file 41372_2024_2046_MOESM1_ESM.tif]

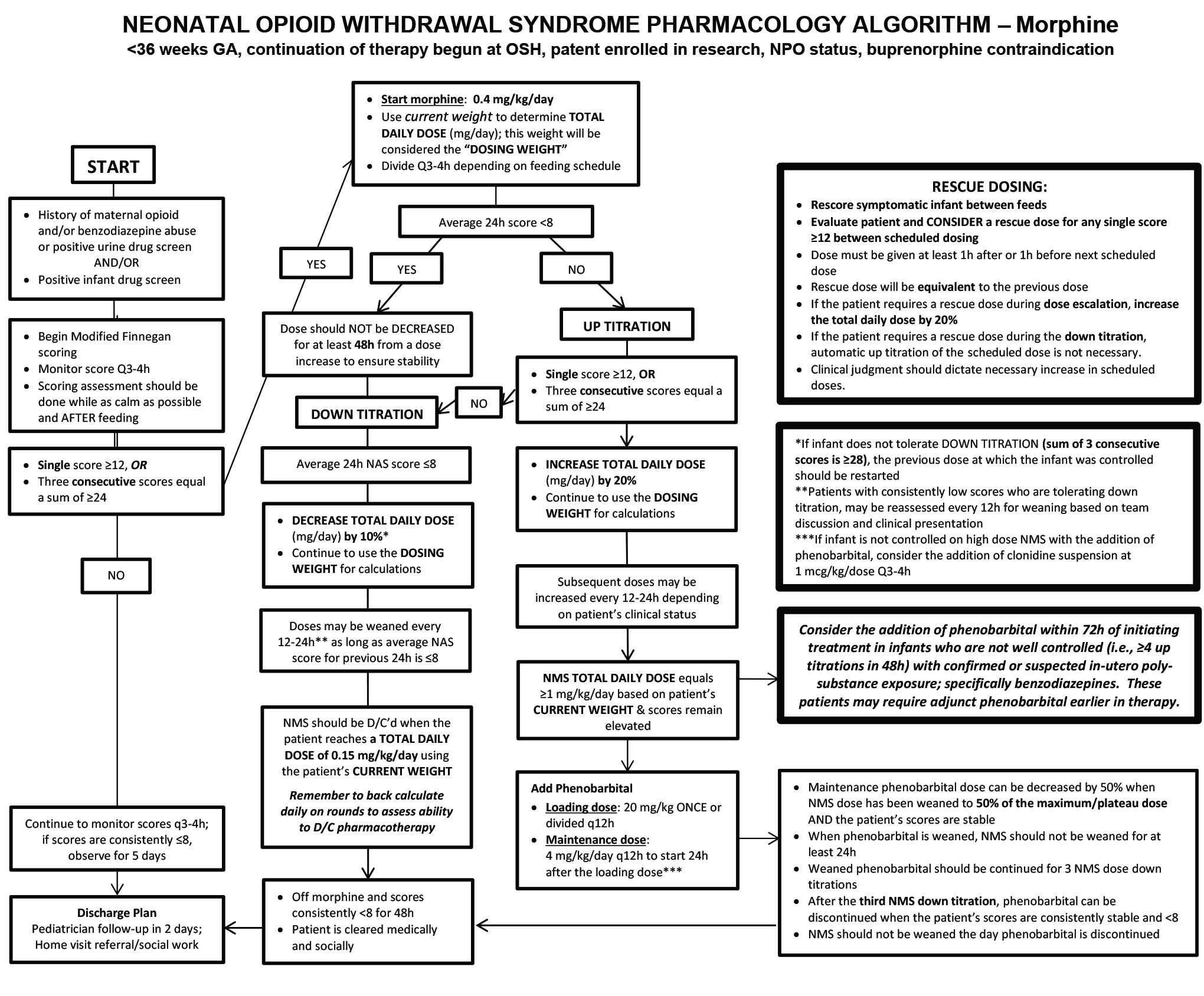

Supplement: Supplementary file 2 — Figure S2. Institutional protocol for management of pharmacotherapy for NOWS using morphine. [file 41372_2024_2046_MOESM2_ESM.tif]
